# Harnessing Artificial Intelligence for Health Message Generation: The Folic Acid Message Engine

**DOI:** 10.2196/28858

**Published:** 2022-01-18

**Authors:** Ralf Schmälzle, Shelby Wilcox

**Affiliations:** 1 Department of Communication Michigan State University East Lansing, MI United States

**Keywords:** human-centered AI, campaigns, health communication, NLP, health promotion

## Abstract

**Background:**

Communication campaigns using social media can raise public awareness; however, they are difficult to sustain. A barrier is the need to generate and constantly post novel but on-topic messages, which creates a resource-intensive bottleneck.

**Objective:**

In this study, we aim to harness the latest advances in artificial intelligence (AI) to build a pilot system that can generate many candidate messages, which could be used for a campaign to suggest novel, on-topic candidate messages. The issue of folic acid, a B-vitamin that helps prevent major birth defects, serves as an example; however, the system can work with other issues that could benefit from higher levels of public awareness.

**Methods:**

We used the *Generative Pretrained Transformer-2* architecture, a machine learning model trained on a large natural language corpus, and fine-tuned it using a data set of autodownloaded tweets about *#folicacid*. The fine-tuned model was then used as a *message engine*, that is, to create new messages about this topic. We conducted a web-based study to gauge how human raters evaluate AI-generated tweet messages compared with original, human-crafted messages.

**Results:**

We found that the *Folic Acid Message Engine* can easily create several hundreds of new messages that appear natural to humans. Web-based raters evaluated the clarity and quality of a human-curated sample of AI-generated messages as on par with human-generated ones. Overall, these results showed that it is feasible to use such a message engine to suggest messages for web-based campaigns that focus on promoting awareness.

**Conclusions:**

The *message engine* can serve as a starting point for more sophisticated AI-guided message creation systems for health communication. Beyond the practical potential of such systems for campaigns in the age of social media, they also hold great scientific potential for the quantitative analysis of message characteristics that promote successful communication. We discuss future developments and obvious ethical challenges that need to be addressed as AI technologies for health persuasion enter the stage.

## Introduction

### Background

Crafting a successful health message involves a mix of art and science. On the one hand, decades of research in linguistics and communication science provides numerous insights into coherent sentence structure, effective value propositions, and other language-specific factors that promote attention, memory, and engagement [1,2]. On the other hand, translating these abstract factors into an appealing, concrete message that could be used in a campaign still requires a leap that must be fueled by human creativity and intuition [3].

Moreover, as larger and longer-term campaigns usually require a multitude of diverse messages, message creation represents a resource-intensive bottleneck. Although computers are often able to increase efficiency related to message development (ie, information gathering, collaborative environments, and graphic designs), the task of message creation was traditionally beyond their scope. Until a few years ago, computers could analyze a sentence and flag errors; however, they were not able to synthesize a meaningful new sentence. However, the latest advances in machine learning (ML) have equipped computers with the ability to generate language for messages that appear natural and readily comprehensible to humans. This work is highly relevant to health communication in general and campaigns in particular as it could be connected to the task of campaign message generation. Specifically, there is the possibility that language generation methods might help in creating and optimizing messages; however, as there has been little contact between the fields of health communication and language generation, more work is needed to examine this possibility. In this paper, we ask, “How feasible is it to automatically generate on-topic messages that could potentially promote awareness about specific health issues?”

In the following section, we first review how the internet and social networking sites have become part and parcel of health communication. Next, we present the health issue of folate or folic acid (FA) as our test case and highlight the need for campaigns to promote FA awareness. We then introduce recent studies on natural language generation (NLG). This leads to a study in which we use a data set of FA-related social media messages to train a *message engine,* which then generates hundreds of new messages about this topic. We evaluate the clarity and quality of these artificial intelligence (AI)–generated messages compared with human-generated content via a web-based study.

### The Potential of Social Media Communication Campaigns to Raise Awareness About Specific Health Topics

Social media has become a key component of communication campaigns [4]. This development has enabled new forms of health communication that are more direct and engaging for users. Social media–based messaging has also led to unprecedented opportunities for optimizing and effectively delivering information to the masses via computationally heavy approaches such as A/B-testing, recommender systems, and targeting receiver characteristics or social network positions [5-10]. Social media can diffuse messages widely across the globe and deeply into interpersonal networks [11,12].

The role of social media within the health communication landscape is still evolving; however, almost all health campaigns have embraced social media as cost-effective and highly scalable channels for raising and sustaining public attention [4,13]. Specific health issues that are affected by a chronic lack of awareness can benefit substantially from social media awareness campaigns. This is perhaps most prominently demonstrated by the success of the amyotrophic lateral sclerosis ice water bucket challenge, which brought substantial public awareness to the disease of amyotrophic lateral sclerosis and encouraged donations to research.

Raising awareness and providing basic information is a critical first step toward prevention, considering that all health communication theories posit that if people are unaware of a specific health risk, they will not take preventive action [14]. Of course, many complex health behaviors involve factors beyond awareness and education, such as shifting norms and attitudes or persuading target audiences to engage in specific behaviors [3,15]. However, for some selected health problems, awareness and knowledge deficits can be the primary campaign goals [16,17], and for many others, raising awareness or keeping the issue on the public agenda [18] is at least a secondary goal. Thus, although we are not claiming that raising awareness is a cure-all solution, we consider it a critical first step for any message generation system.

### The Case of FA Awareness

Simply raising awareness and providing essential knowledge can go a long way for prenatal health. Many people who are pregnant are intrinsically motivated to adhere to health recommendations if they know them, as can be measured via self-report and behavioral indicators, such as smoking quitting attempts, reduction in drinking, and changes in exercise and nutrition behaviors [19-21]. This includes eating a folate-rich diet (to minimize the risk of neural tube defects [NTDs]) or avoiding rare meat (risk of toxoplasmosis) and certain cheeses (to reduce the risk of listeria infection). However, awareness about FA and knowledge about FA-rich diets among women of childbearing age remain too low [22-24]. This is problematic as most pregnancies are only noticed after NTDs occur, such that once people learn about effective prevention behaviors during, for example, a physician’s visit, it may be too late [25,26]. Therefore, the issue of FA awareness will serve as a proof-of-concept example to demonstrate the potential of AI-generated messages that could potentially be used to raise awareness by providing a steady feed of on-topic but novel messages in long-term health campaigns.

*Folate* is a vitamin that is required for the body to build cells [27]. Many fruits, vegetables, and other natural foods contain folate, and the synthetic form, *FA*, is used as a dietary supplement or food additive. A folate or FA deficiency during early pregnancy can lead to severe embryonal NTDs [28]. Thus, the World Health Organization and the Centers for Disease Control and Prevention (CDC) recommend that all women of childbearing age consume 400 µg of folate per day [29,30].

Lack of awareness about FA represents a problem that is, at least to some degree, preventable via health communication and education [22,31-33]. As argued above, most people who are pregnant are motivated to achieve FA supply but will only be able to follow the guidelines if they are aware of them in the first place. Moreover, the recommended steps are relatively easy to follow for many people. However, that is not to say that by simply raising awareness, all positive downstream effects would follow. As with most health behaviors, they are embedded in a biopsychosocial context, requiring, for example, availability of food or FA supplementation, cultural factors, and so forth. However, the basic problem constellation of lack of awareness, paired with a relatively high spontaneous motivation and high self-efficacy and response efficacy, suggests that mass media health campaigns are a promising strategy. Indeed, several previous studies support that FA-related campaigns can produce positive effects [22,31,33].

### New Challenges for Social Media Communication Campaigns

The key benefit of mass media campaigns on social media is that they can quickly disseminate messages into the homes of millions. Moreover, social media has made it much easier to reach specific audience demographics and keep track of relevant outcomes, such as whether messages are seen, shared, or commented on [3,4].

However, although campaigns are a highly scalable tool, conducting a successful campaign is still far from trivial and requires substantial monetary investment and sustained effort over a longer period [3,15,34]. When it is properly conducted, mass communication is highly cost-effective compared with other approaches [35,36], and most campaigns do not achieve high levels of exposure over a sustained period [35]. For instance, most campaigns only achieve approximately 40% exposure in their target audience [37], which naturally reduces their success as communication effects logically require that messages are seen in the first place [38]. Moreover, in the days of print, radio, and television campaigning, many campaigns comprised only a limited number of messages that were switched at a relatively slow rate (eg, weekly or monthly), if at all. Although the more professional campaigns nowadays feature *feeds* with dozens of messages, if not more [4], maintaining such an effort is very costly and requires dedicated personnel, formative processes, and summative evaluation throughout [39,40]. In summary, campaign creation and maintenance is an effortful business.

However, even campaigns that are executed skillfully have difficulties in reaching their audience. The low 40% exposure rate mentioned above came from a study published in 2004; however, since then, the internet has further exacerbated the competition for attention [41-43]. Specifically, the very nature of today’s attention economy on social media requires that health communicators update content frequently. Otherwise, algorithms will downvote the content and make it less likely to be seen by the target audience [43,44]. Similarly, on the side of the audience, switching behavior and searching for novel information are very widespread [45]. In summary, the logistic effort needed to create campaign messages and ensure their constant dissemination, as well as the algorithmic and user-sided information selection decisions, pose challenges for maximizing the potential of health communication campaigns.

Overall, this situation invites new approaches that could help health communicators and practitioners create a large number of awareness messages, which could then be automatically scheduled to ensure a constant and variable feed of appealing and timely messages. The following section introduces how recent developments in NLG, a subfield of machine learning or AI research, offer a potential solution to message development and dissemination limitations.

### The Potential of Language Models to Generate Domain-Specific Health Messages

Advances in natural language processing have made it increasingly possible to generate coherent messages [46]. Although enthusiasm and skepticism about using computers for text generation have waxed and waned for decades [47], the advances in the past decade have been particularly impressive as the quality of computer-generated texts is now at a level that makes it often indiscriminable from human-written text [48,49].

A model that attracted substantial public attention is the *Generative Pretrained Transformer-2* (GPT-2) [50]. In brief, GPT-2 is a deep learning–based ML model that performs expertly across several language-related tasks, such as text translation and summarization, question answering, and text generation [51,52]. Approximately 40 GB of data from >8 million webpages were used to train the basic model. GPT-2 comes in 4 sizes ranging from 124M, 355M, 774M, and 1.5B parameters. Humans generally find the output of GPT-2’s text generations authentic and interesting. Notably, the model is publicly available and can be adapted to many text-based tasks, such as summarization, question answering, or generation.

Pretrained language models can be fine-tuned to specific domains [53]. Fine-tuning is a form of transfer learning in which an ML model trained on domain-general data is retrained on further domain-specific data to adapt to its particularities. The possibility of using such fine-tuned language models to generate domain-specific text has already been demonstrated across different disciplines [54,55]. However, we are not aware of any effort to examine this in the context of health communication. Thus, the question is whether fine-tuning GPT-2 to the domain of FA messages will enable it to generate new messages that are of sufficient clarity and quality to be useful for a potential social media health campaign.

### Present Study

This research examines the capability of language models to generate realistic messages about FA, which could serve as suggestions for a potential health campaign. Furthermore, we ask whether this is realistic in the context of public health communication, a situation often characterized by a lack of funds and computational resources. In brief, we use messages, or tweets, from the popular message-sharing platform Twitter to fine-tune a GPT-2 model. Although the same approach can be used with other social media platforms, Twitter offers a relatively straightforward, mainly text-based message format with a 280-character limit and easy access to existing messages, making it the most promising candidate for piloting such a system. After downloading messages and training the message engine, we use the fine-tuned model as a *message engine* to generate a large number of new FA-specific tweets. We then examine the characteristics of the generated messages to identify the preconditions for success and the current limitations of these generated messages. Finally, we wanted to know how AI-generated messages would be compared against human-generated messages. To this end, we conduct a web-based study in which human judges evaluate the AI-generated and human-generated tweets in terms of clarity and quality.

## Methods

### The FA Message Engine: Overall System Description

#### Overview

**Figure 1:**
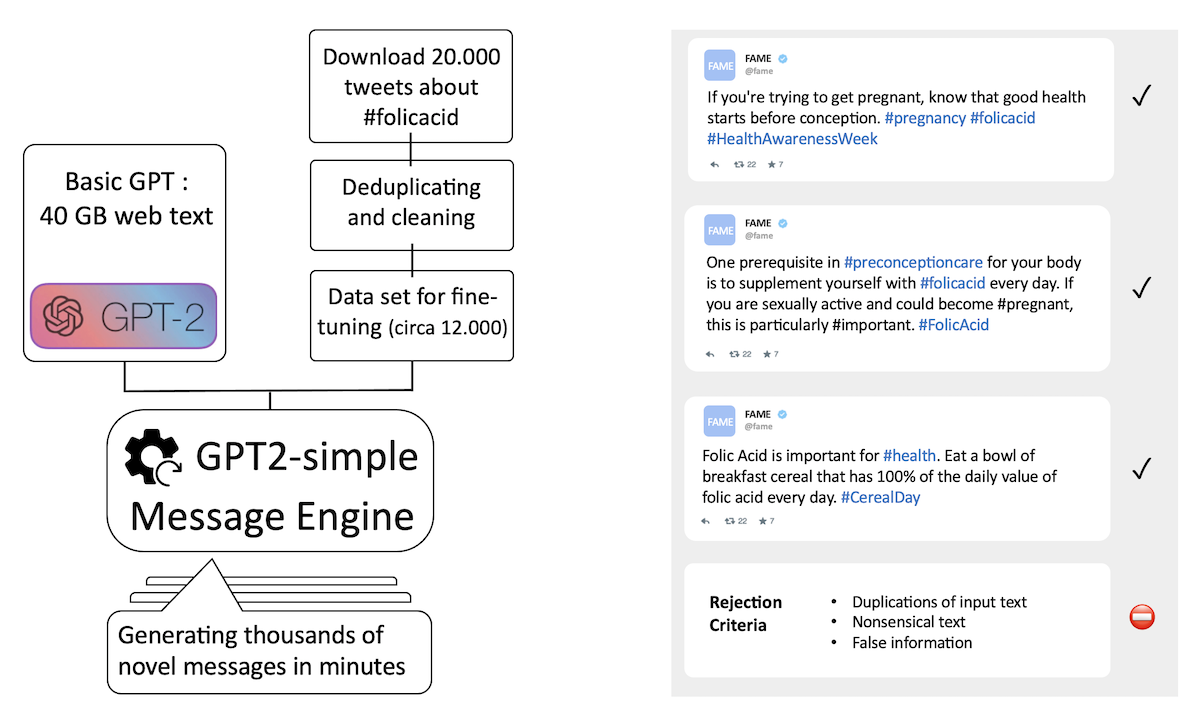
The left panel provides a schematic overview of the message engine construction and message generation workflow. The right panel illustrates a few examples of candidate messages. GPT: Generative Pretrained Transformer; GPT-2: Generative Pretrained Transformer-2.

In this study, we harnessed the latest advances in AI to build a system that can generate a near-infinite number of health messages to promote FA awareness—the FA Message Engine. What will further be called the *message engine* is essentially an instantiation of the GPT-2 simple system, a Python package dedicated to fine-tuning OpenAI’s GPT-2 text generation model [56]. We used the medium-sized GPT-2 model (355M hyperparameter versions trained on 40 GB of web text) and fine-tuned it with a data set of autodownloaded tweets about *#folicacid*. The resulting model was used to generate new messages (Figure 1).

Our specific steps are discussed in the following sections.

#### Scraping Tweets for Model Retraining

To obtain a data set to fine-tune the model, we used the Twitter Intelligence Tool [57] to scrape a large number of tweets that mentioned *#folicacid* in their text. Specifically, we downloaded 25,304 tweets posted between 2010 and 2020 that mentioned *#folicacid* and extracted the raw text of the tweets. After removing duplicates, non-English tweets, and tweets that mainly promoted nutritional supplements (stopwords: buy, order, or sale), we ended up with a data set of 11,311 unique tweets for fine-tuning.

#### Fine Tuning the GPT-2

After downloading and cleaning the messages to create a data set for fine-tuning, the next step involved preparing and retraining the GPT-2 model. Specifically, we submitted the data set for fine-tuning to retrain the 355M GPT-2 model with recommended default settings of 2000 training steps and a learning rate of α=.0001. Fine-tuning was accomplished via Jupyter Notebook running Python 3 on a computer equipped with a graphics processing unit and executing the gpt2.finetune()-Method from the gpt-simple package [56]. On a standard graphics processing unit–equipped computer, fine-tuning a model of this size takes approximately 1 hour. We also conducted pilot experiments with other model sizes but chose to put only the medium-sized 355M GPT-2 model for a user test. Larger models require advanced hardware, whereas the medium model can work with most cloud-based computing services available to end users. Larger models are also not recommended for generating short text messages, such as tweets. After training, the fine-tuned model, which constitutes the *message engine*, was saved to the disk.

#### Generating Candidate Tweets via the Message Engine

We used the *message engine* with a default temperature setting of *t*=0.7 to generate 1000 new tweets. Temperature settings influence the randomness of the textual output, with a lower temperature being less random. As for model size, we conducted pilot tests with different temperature settings but noted that higher settings (*t*=1.0) produced very incoherent output, and low settings (*t*=0.3 and *t*=0.5) led to text that was very close to the training data. Given that our goal was to test the engine’s output in humans in terms of clarity and quality, we deemed it worthwhile to conduct user testing for this setting, which is also the recommended default setting as per gpt2-simple’s documentary [56].

### Evaluating AI-Generated Messages: Web-Based Study

To evaluate the clarity and quality of tweets generated by the *message engine* against human-generated tweets, we performed a web-based study. The procedure was devised based on the emerging guidelines for evaluating NLG studies [58] and is described in the following sections.

#### Message Selection

From the 1000 AI-generated tweets, we drew a random sample of 60 tweets. Next, a human editor curated these tweet suggestions and compiled them into a set of 30 tweets for the web-based study. The human editor rejected AI-tweet suggestions if they contained duplications from the input data, false information according to CDC guidelines, or problematic advice (see the following section for details). A second human curator confirmed this selection without contradictions.

In parallel, we drew a random sample of 30 tweets from a pool of >10,000 real-life tweets. This strategy was chosen as it is not feasible to evaluate thousands of tweets and as it most likely mimics how practitioners would use such a system [49]. Thus, this procedure yielded 2 sets of 30 tweets each—30 AI-generated messages that came from a pool of 60 randomly drawn samples and 30 human-generated messages from Twitter.

#### Participants

We recruited 150 young adults from a web-based pool at a large Midwestern university to evaluate these messages in terms of clarity and quality. Study participants received course credit as reimbursement for completing the short survey, which lasted approximately 20 minutes and was approved by the local institutional review board. Of the 150 young adults, after excluding data from participants who did not finish the survey or responded unrealistically fast and clicked through the survey, we ended up with a data set of 129 (86%) respondents (mean age 20 years; range 18-28 years). The sample was predominantly female (96/129, 74.4%). Although this sample was not intended to be representative of the population, our participants clearly belonged to the audience of a potential FA awareness campaign. Moreover, given that the goal was to evaluate message clarity and quality rather than message effects on attitudes or behavior, this sample is sufficient for this purpose.

A power analysis suggested that a sample of approximately 100 raters was sufficient to detect a small-to-moderate effect in terms of the mean difference in evaluations of AI-generated and human-generated tweets (1-β=0.9; α=.05; *dz*=0.3) [59]. Moreover, message evaluation studies suggest that evaluations of individual messages stabilize after averaging data from approximately 25 to 30 raters per message [60], which we surpassed with this sample size.

#### Procedure

The survey was administered via Qualtrics software (Qualtrics International), and participants were asked to evaluate all messages regarding clarity and quality. Participants were told that the study’s goal was to examine human evaluations of Twitter messages about FA or folate, such as whether they considered the messages adequate to raise awareness or educate audiences about this health issue. The test messages were presented randomly, and participants were unaware of whether they came from the pool of AI-generated or human-generated messages. Each message was evaluated on 2 questions, 1 focusing on message clarity (“Please evaluate this message in terms of whether it is clear and easy to understand.”) and 1 on message quality (“How much do you agree that the content and quality of this message is appropriate to increase public knowledge about folic acid?”). Answers were collected using a 5-point Likert-style response format (*very clear* and *very unclear* and *strongly agree* and *strongly disagree*). At the end of the survey, participants were debriefed about the study’s purpose and provided a link to the CDC’s website for the most up-to-date information on FA.

### Evaluating AI-Generated Messages: Computational Analyses

In addition to inspecting the AI-generated messages and performing a web-based evaluation study, we conducted several computational analyses. Specifically, we computed n-grams and inspected their distribution between AI- and human-generated messages, including visualizations as word clouds. Next, we performed topic modeling analyses to gain additional insights into the semantic structure. Topic modeling is a prominent method for identifying health topics in social media [61] or subtopics within a given health domain [62-64]. Specifically, we used the topicmodels package [65] within the R statistical software to compute the latent Dirichlet allocation topic models [66]. Finally, we assessed the semantic similarity of individual messages via the sentence-transformers package [67]. To this end, we transformed each message into a sentence embedding and compared different messages via cosine-vector similarity.

## Results

### Overview

We found that the fine-tuned GPT-2 model can act as a *message engine* by creating grammatically correct, coherent, and novel messages centered on the topics of FA, healthy nutrition, and pregnancy. In the following sections, we will first describe the insights gained during the overall procedure and qualitative characteristics of the generated output, followed by the web-based evaluation study’s quantitative results.

### Feasibility of the System and Qualitative Description of the AI-Generated Messages

Our overall research question focused on whether it is possible to fine-tune a language model such as GPT-2 to a specific health domain to build a *message engine.* The answer is that it is possible. As can be seen from the sample output in Figure 1, the *FA Message Engine* was able to generate 1000 tweets within a matter of minutes, most of which resembled authentic web-based messages in style and content.

We next asked whether training such a system is realistic in the context of public health, where computational resources and specialized coding skills are scarce. The answer to this question is that it is feasible and surprisingly easy to implement. Although developing the scraping, cleaning, and training procedure took some time, now that the system is set up, it can be replicated with little effort. For instance, if we wanted to replace the topic of *#folicacid* with any other health issue, this can be done in >1 hour. The system is also relatively accessible, even to novice users, as long as they are able to execute Python notebooks. Such skill requires only little training, and it would be possible to build a user interface for the system such that the user only enters the topic or search term (eg, *#folicacid*) and, after fine-tuning and generation, receives a sample of 60 message suggestions.

Most critically, we were interested in the characteristics of the generated messages, to which we turn next.

First, we note that the vast majority of the AI-generated tweets appeared natural and contained many elements of the original input tweets that were scraped from Twitter. For instance, the system uses hashtags that co-occurred with the search term *#folicacid*, such as *#pregnancy, #vitamin, #foodfortification #folicacidawarenessweek,* or *#eathealthy*. Second, as with hashtags, the system also tagged accounts that appeared in the input data, such as @*CDC* or @*NHS* (note that by eliminating these accounts from the input data, such information can be suppressed if not wanted).

Another observation is that most of the generated tweets were rather engaging, enthusiastic, or upbeat. This impression may again arise as the input tweets contained elements such as prompts with exclamation marks (“Eat healthy *now*!” and “*Go Folic! Visit [URL]!*”) or encouragement, all of which could be interpreted as *cues-to-action* or attempts to raise *self-efficacy* according to the Health Belief Model [68]. This characteristic is likely as GPT-2 was trained on outgoing links with high so-called *karma scores* [50], thereby selectively emphasizing the language that web-based audiences found interesting and engaging.

Beyond resembling the linguistic style and platform-specific cues that are characteristic of today’s Twitter environment (eg, upbeat language and hashtags), we observed that the AI-generated tweets reflected the input data’s topic distribution. For instance, input tweets could be categorized into several topical clusters, such as nutritional needs during pregnancy, the link between FA and NTDs, political advocacy for mandatory food fortification, and so forth. Most AI-generated messages could also be categorized into coarse topic clusters. Additional results and visualizations of n-grams, word clouds, and results from topic modeling can be found in Multimedia Appendix 1 [65-67,69-73].

Although the overall system and procedure proved feasible, and the quality of many messages appeared comparable with human-generated messages, we made several observations that point to current limitations.

A simple observation is that the system sometimes parrots the training data; that is, it contains either duplicates of raw tweets or specific formulations that appeared in the data set used for fine-tuning (eg, “If you are trying to get pregnant...” and “Thinking of trying for a baby...”). This issue is well-known and follows logically from the fact that language models are essentially giant statistical association machines, which will learn the information contained in the input data. From an intellectual property perspective, this issue can raise questions about the copyright of the generated output. However, in practice, it is easy to sort out such parrot generations through human supervision, n-gram matching, or paraphrase detection algorithms.

A second limiting observation is that even when not directly parroting the training data, many tweets are still *close to* individual input messages, for example, by mixing formulations such as *trying to get pregnant* and *thinking of having a baby* with various combinations like *start taking #folicacid* or *know that good health starts before conception*. This points to mere reformulations and permutations that are not very creative. Again, this represents a direct consequence of the way natural language models work and is thus not necessarily a severe limitation. In fact, variations of a common message on a health topic may improve a campaign’s reach by preventing the content from being downvoted by algorithms that select for novelty. Slight variations may also be beneficial in improving existing messages by making them briefer or more engaging, and it is well-known that repeated exposure to messages improves awareness and retention [38,74-76]. Rather than a limitation, this reformulation strategy of message generation can help to more optimally exhaust the space of possible effective messages. Nevertheless, it is clear that mere rewording represents only a minor achievement in message creation.

A third observation is that some generated messages contained false statements about which foods contain which amounts of FA or what medical defects might occur. Although such tweets are easy to spot in practice, this is an actual limitation. A total of 2 factors may underlie such behavior. First, if the input data contain false or problematic health claims, which are pervasive on social media, then the system will learn them. In this case, the system should not be blamed; however, the curation of the data set for fine-tuning should be optimized. However, more critically, the state of the art of current language models implies that they will simply generate tweets that sound linguistically coherent but may not make sense. We have discussed this issue in the *Discussion* section*,* where we suggest advancements to the system.

These results suggest that human curation and supervision of AI-generated tweets are necessary for practical use cases. Thus, a campaign manager or team would need to monitor the retraining process and eliminate problematic content, which is also what we opted for to select tweets for the web-based evaluation study.

### Quantitative Comparison of AI- and Human-Generated Messages

**Figure 2:**
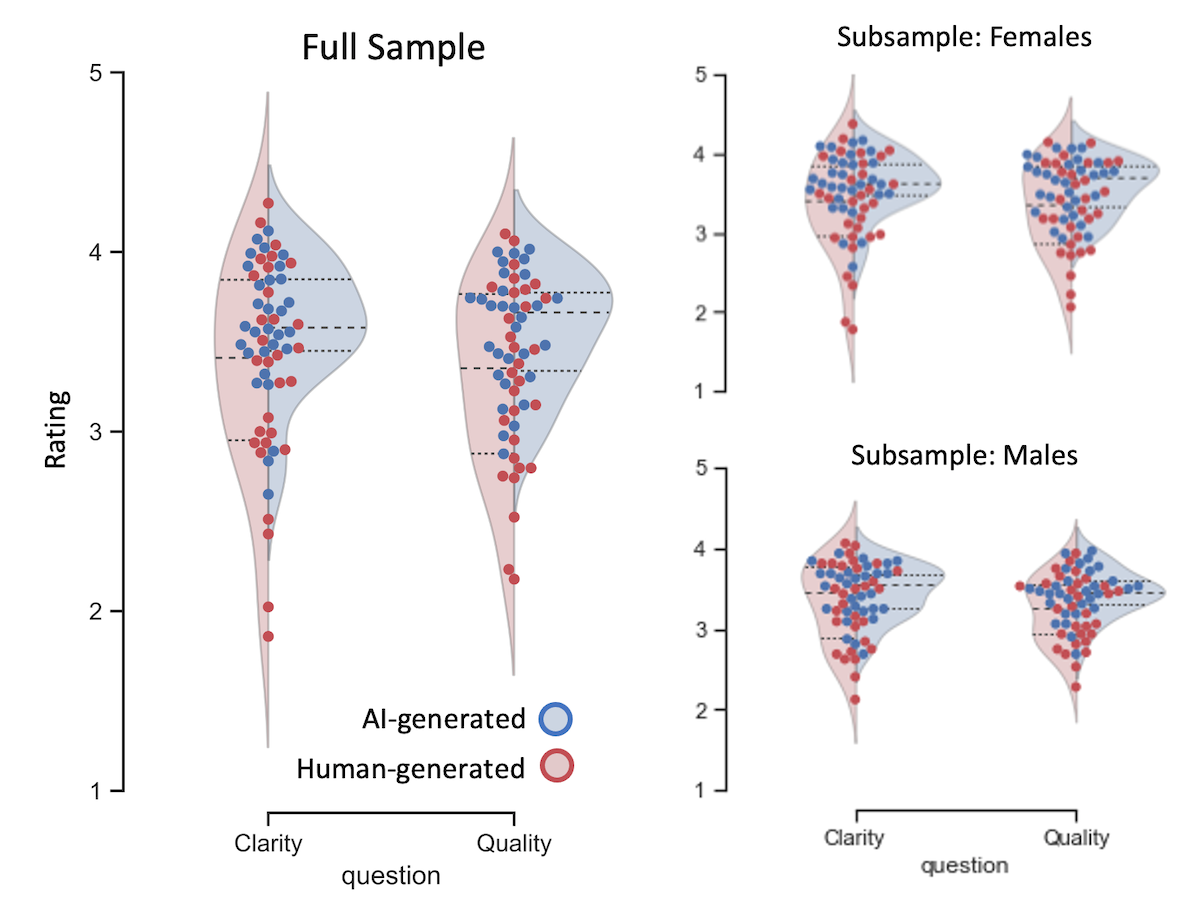
30 artificial intelligence–generated tweets (blue) and 30 human-generated tweets (red) were evaluated in terms of perceived clarity and quality on a 5-point Likert-style scale. The results revealed very similar evaluations and minor average differences, with a considerable spread within each category. The right panels show analyses separated by gender. AI: artificial intelligence.

Table 1 shows the results of comparing AI-generated and human-generated tweets in terms of overall clarity or quality. Figure 2 illustrates the results graphically and provides further distributional information as well as analyses by subgroups. As can be seen, overall, the messages were rated as relatively clear and easy to understand, and participants found that their content and quality were appropriate for increasing public knowledge about FA (>3 on a 5-point scale).

**Table 1:** Means (SDs) from the web-based survey. Scores for message clarity and quality evaluations for 30 AI^a^- and 30 human-generated messages are shown, respectively.

Evaluations	AI-generated messages, mean (SD)	Human-generated messages, mean (SD)	*t* test (*df*)	P value
Clarity	3.58 (0.36)	3.34 (0.6)	1.97 (58)	.05
Quality	3.57 (0.32)	3.3 (0.53)	2.34 (58)	.02

^a^AI: artificial intelligence.

Statistical analysis revealed that the AI-generated and human-generated tweets were not rated as different in terms of how clear and easy they were to understand (*t_clarity_*=1.97; P=.05). A small but statistically significant difference was found in the quality dimension (*t_quality_*=2.34, P=.02). However, as can be seen from Figure 2, these mean differences were small in light of the variability, and thus, the effect was of a small size. We also zoomed in on the women participants’ subgroup as the topic may be more relevant to them or become more relevant in the future with respect to pregnancy. As can be seen from Figure 2, the results were robust, and both subgroups (women and men) exhibited essentially the same pattern of results. However, we noted that, given the sample of college students, the topic of FA might not be very relevant to them, although there are also other health benefits of FA beyond the prevention of birth defects.

Next, we performed analyses at the level of the individual messages. As shown in Figure 2, the differences for both clarity and quality between individual messages were larger than the differences between categories (human- vs AI-generated). Indeed, performing item-wise analyses in which we compared ratings for single messages across raters using dependent-sample tests (because the same raters evaluated all messages) revealed that many messages were rated consistently higher than others. This pattern emerged both within human-generated and AI-generated messages, as well as across categories. Thus, many AI-generated messages were rated much higher than random human-generated messages.

Overall, we took these results as evidence that the *message engine* generated tweets that human raters evaluated as mainly equivalent to real Twitter messages.

### Computational Analyses

In addition to the analyses of content (n-grams and topic modeling) and the human evaluation of clarity and quality, we wanted to examine the generated messages using computational methods. Specifically, we compared the 60 messages (30 AI-generated and 30 human-generated) using sentence Bidirectional Encoder Representations from Transformers (BERT), a modification of the pretrained bidirectional encoder representations from transformers model, to derive semantic sentence embeddings, which we then compared using cosine-similarity [67].

We found that across all messages, the average similarity was *s*=0.35. Within the 30 AI-generated messages, the average similarity was *s_AI_*=0.37, and the average similarity between the 30 human-generated messages was s_Human_=0.34. The average similarity between AI versus human messages was *s_AI_* versus *s_Human_*=0.35. Testing for differences between these computational indices of semantic similarities revealed no significant differences in any comparison (AI vs human and within- vs across-classes; all P>.08; for further details, see Multimedia Appendix 1 [65,66]). These results suggest that the sample of AI-generated messages is semantically similar to the sample of human-generated messages.

## Discussion

### Principal Findings

This study examined whether AI message generation technology can create candidate messages for use in social media health campaigns that focus primarily on raising awareness or increasing knowledge. We found that by retraining a GPT-2 model with thousands of tweets about FA, it is possible to build a *message engine* that can generate novel tweets, which could become part of an actual campaign. Human raters perceived these tweets as broadly similar in terms of clarity and quality to real-world messages. These results suggest that AI-assisted *message engines* could support campaign staff to create more efficient and possibly more effective campaigns for topics that are suitable for awareness-based messaging.

To our knowledge, this study is the first to demonstrate the potential of automated message generation in the context of health communication. Our results are generally positive, suggesting that the *FA Message Engine* can serve as a starting point for more sophisticated AI aides for message generation. Such systems can automatically offer thousands of messages that mimic the style and reflect the substance of existing health messages. Given that message creation is a resource-intensive bottleneck, we see significant application potential for such a system as a catalyst for human creativity [77].

Building a message engine for the topic of FA proved to be surprisingly easy. Our system made use of available tools [56] and could thus be transferred to contexts other than the issue of FA. Although this work did not intend to provide such a general purpose system for end users, it would be only a small step to deploy it as a web application as a turnkey solution.

Although the results of the web-based study demonstrate that the system output achieves good results, and the clarity ratings of AI-generated tweets are even significantly higher, we emphasize that our comparison strategy does not warrant the conclusion that AI-generated messages are superior to human-generated messages. Specifically, we compared a selection of 30 AI-generated messages against a sample of 30 real tweets, which were randomly drawn from a pool of >10,000 tweets. We opted for this procedure as our goal was to test the feasibility of AI-assisted message generation, which is the most realistic use case. In the following sections, we have discussed the significant limitations that currently prevent such a system from operating independently. However, the pool of human-curated AI-generated messages performed on par with or better than the standard tweets, and the analysis of semantic similarities did not reveal any difference. Thus, our approach suggests a simple strategy that might improve the quality of web-based content while saving the time and money of health communication practitioners.

Beyond the practical potential of such a *message engine* in the age of social media, the approach also offers considerable scientific potential. In particular, AI-based message engines might strengthen strategies to analyze message characteristics that underlie successful health communication [78-80]. In its current form, users of the message engine cannot influence the generated text’s characteristics other than by what is fed in with the fine-tuning data set.

However, the natural language processing community [55,81,82] strives to gain more control over how the text is generated, and we see this as a promising next step. In particular, a limitation of the current system is that it does not incorporate any theory-based message design principles [1,83], such as barriers, cues to action, and norm or threat appeals. The fact that the current system learned to include some theory-compatible features, such as cues to action, shows promise in this regard; however, a more systematic approach is needed [84-86].

Ideally, this could then set off a virtuous cycle in which one could, via rapid iterations, gather feedback about specific message characteristics that are associated with targeted outcomes (eg, attention, awareness, and message sharing) and thus more clearly identify the message characteristics that facilitate individual outcomes [87]. As these characteristics become more accessible by linking objective message properties to large-scale outcomes, we might expect profound theoretical contributions from this otherwise applied system [88].

Along these lines, the most promising research direction is to fine-tune the fine-tuning process. We simply used the medium-sized GPT-2 model and fine-tuned the model with a set of tweets that were minimally screened. However, as with any manufacturing process, the quality of the input data determines the output. Thus, by fine-tuning the engine with often mediocre tweets, the AI-generated tweets were likely less potent than they would have been with a better training set.

In the future, we envision that one could curate a pool of high-quality tweets to serve as grade-A training material. An option, analogous to the strategy of training the GPT-2 base model with relatively more engaging text content, is to select only those tweets about *#folicacid* that have been retweeted or liked. Another option is to bootstrap messages by having domain experts reword or craft theory-based examples. However, a challenge for this strategy is that fine-tuning requires large amounts of text—a few hundred examples are not enough. Overcoming this challenge is feasible with a large pool of quality input data. In addition, such a message pool could be used to train message engines for domains other than the narrow issue of FA.

We conclude this section by emphasizing again that the primary use case of such systems lies in boosting awareness for selected health problems where awareness is lacking or waning. At this point, a message engine system does not yet solve trickier health communication problems, such as the habitual nature of many negative health behaviors, addressing the socioecological embeddedness of such behaviors, or how to change health-related attitudes [89]. In principle, we see no reason why such systems could not be expanded to contexts beyond social media, especially as reliance on voice assistants such as Amazon’s Alexa, Alibaba’s AliGenie, or Apple’s Siri for information increases. However, the *message engine* presented here is primarily intended for mass communication about public health issues that are affected by low awareness. For such health issues, we envision that this system can improve the cost/benefit ratio and overcome the *message-creation bottleneck* to avoid web-based content from being algorithmically downvoted as it is considered not fresh, dull, or unengaging.

### Limitations, Risks, and Avenues for Future Research

This study demonstrates a positive application of NLG technology; however, some risks and limitations are worth mentioning.

A very basic limitation is that our focus was on demonstrating the feasibility of a message engine to generate messages that could potentially become part of a campaign; however, we did not actually conduct such a campaign. Thus, although we are confident that we showed that the generated messages—after going through the human content curation process—are on par with human-generated *baseline* messages, we did not actually show that these messages improved public health and especially not with regard to more distal outcomes such as attitude change and behavior. This should be the topic for future research.

Similarly, we note that our sample comprised college students who were not intended to be representative of the larger population. However, given that our focus was on evaluations of message clarity and quality rather than more idiosyncratically defined responses, this sample seems appropriate. This is also underscored by the fact that evaluations were highly consistent across subgroups of women and men raters. Nevertheless, future work on, for example, message effects on attitudes, beliefs, and other variables beyond basic clarity and quality should also focus on outcomes in specific health audiences, such as people who intend to have a child.

Regarding broader implications and risks, recent events in the political domain have highlighted the danger of algorithmic bots deployed to create or spread misinformation [90-93]. Several malicious actors seem to be using natural language generators to produce fake or divisive messages; thus, several empirical studies have examined the dangers of using NLG technology to create content that is harmful to society [49,94]. The same problems arise concerning the marketing of products that might harm health or use bots to promote certain brands [95-97].

Our study speaks to these issues by showing that it is also possible that benevolent actors can use NLG methods to promote public health. As with all technologies, risk and benefit are correlated, because otherwise the technology would be abandoned [98]. As such, we hope that our study will help explore the potential of AI as a force for promoting positive outcomes. However, this does not mean that we advocate for a laissez-faire strategy. Instead, a discussion of the ethical consequences of these technologies is needed and ongoing [99,100]. However, the field of health communication seems to be a particularly strong example of how human-centered AI could be used for social good.

Another risk and major limitation of the system is its lack of common sense knowledge. People who are not familiar with NLG technology are sometimes ambivalent about the idea of a *message engine*, finding it both magical and critical. Many also expect the systems to operate in a human-like fashion; however, this is not at all the case. On the surface, the generated tweets have a human-like look and feel to them; however, closer inspection reveals that GPT-2–style language models lack the deeper understanding and reasoning capacity that would be necessary for calling them intelligent.

Indeed, some AI-generated tweets are ludicrous, and others contain false information that is presented as fact [101]. For example, a tweet that emerged with little pretraining material was, “Make sure you drink 4 breads of #folicacid per day!” Such examples reflect a lack of common sense knowledge that one cannot drink bread. These examples arise only as GPT-2, although very sophisticated, ultimately boils down to a statistical association machine that links the domains nutrition and FA but does not have knowledge about fluid versus solid substances. The computer programs’ inability to draw connections between categories or classify knowledge outside the training set has been a fundamental challenge since the early days of AI research [102-104].

This issue becomes particularly sensitive when generations contain wrong medical advice, such as “Take 4 lbs of FA per day!” Again, this reveals that the language model only picks up on statistical regularities in how words are used; however, it possesses no actual knowledge about pregnancy, nutrition, quantities, the developing fetus, and the causal relations between these concepts. These challenges are relatively easy to overcome with human supervision and insight. However, a trickier issue concerns issues in which the underlying knowledge is still evolving or uncertain. Such situations can provide fertile grounds for health myths or speculations about side effects. Such information will enter the message engine if it is present in the training data set, which again emphasizes the need for human curation.

In addition to the lack of domain knowledge about health and biology beyond that provided in the training set, we have already pointed out that the system also has no theoretical understanding of communication and persuasion. The message engine only mimics and varies word use, albeit very eloquently. For a nonnative speaker who has to learn a foreign language for years, this skill may seem enviable; the ability to swiftly come up with 1000 sentences about FA may also impress and help health campaigners who spend hours coming up with 100 new candidate messages. However, the fact that such language models may talk without real understanding also means that they should only be used under the supervision of medical and communication experts. However, this is also true for more human-centric methods, such as focus groups or user-generated content.

Despite these limitations, the underlying technology can be expected to improve rapidly, and health communication researchers are well-advised to keep an eye on these developments. For instance, a successor model to GPT-2 has already been developed [105]. This model, called GPT-3, significantly improves some of the limitations that characterize GPT-2. Together with systems that are capable of generating persuasive arguments, selecting best-matching arguments for specific groups, and several other advances, we anticipate that the field of AI-assisted health message generation will see significant progress over the next decade [65,106-110].

### Conclusions

To conclude, the *message engine* can generate candidate messages for human curators about selected health issues. This is relevant for issues where a lack of awareness is the primary problem, and a rich pool of social media messages is needed. At this stage, human supervision is necessary, and the technology, although very promising for content creation, requires control to select relevant content. Scientifically, this approach may promote a new wave of theoretical insights into the mechanisms of effective health messaging. We foresee that AI-generated messages for health promotion, education, and persuasion will become commonplace. It will be important for health communication researchers and practitioners to develop a strategy to use this technology positively.

## Appendix

Multimedia Appendix 1 Inspection of word frequency, topic modeling results, and analysis of semantic similarity.

## Abbreviations

AI: artificial intelligence
CDC: Centers for Disease Control and Prevention
FA: folic acid
GPT-2: Generative Pretrained Transformer-2
ML: machine learning
NLG: natural language generation
NTD: neural tube defect
