# Supplementary material for: Harnessing Artificial Intelligence for Health Message Generation: The Folic Acid Message Engine
Source: J Med Internet Res. 2022 Jan 18;24(1):e28858. doi: 10.2196/28858 (PMC8808340; doi:10.2196/28858)
Supplement: Multimedia Appendix 1 [file jmir_v24i1e28858_app1.docx]

## **Harnessing AI for Health Message Generation: The Folic Acid Message Engine**

## **Supplementary Materials**

## **Inspection of word frequency**

We began by examining the corpora of Human- and AI generated messages visually, followed by basic analyses of word frequency. The following table illustrates the top-4 uni- and bi-grams in both corpora.


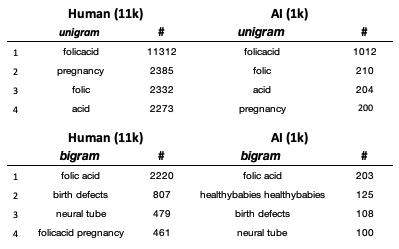


***Supplementary Table 1:*** *Top 4 uni- and bi-grams for Human- and AI-generated messages, respectively.*

Next, we created simple word clouds for the Human- and AI-generated messages, as well as for the selected sample of 60 messages (30 Human- and 30 AI-generated) that were tested in the online study. As can be seen in Supplementary Figure 1, these plots contain many similar word, which confirms that the fine-tuning process succeeded insofar as that the model uses words that were also present in the input data (i.e. Human-generated messages).


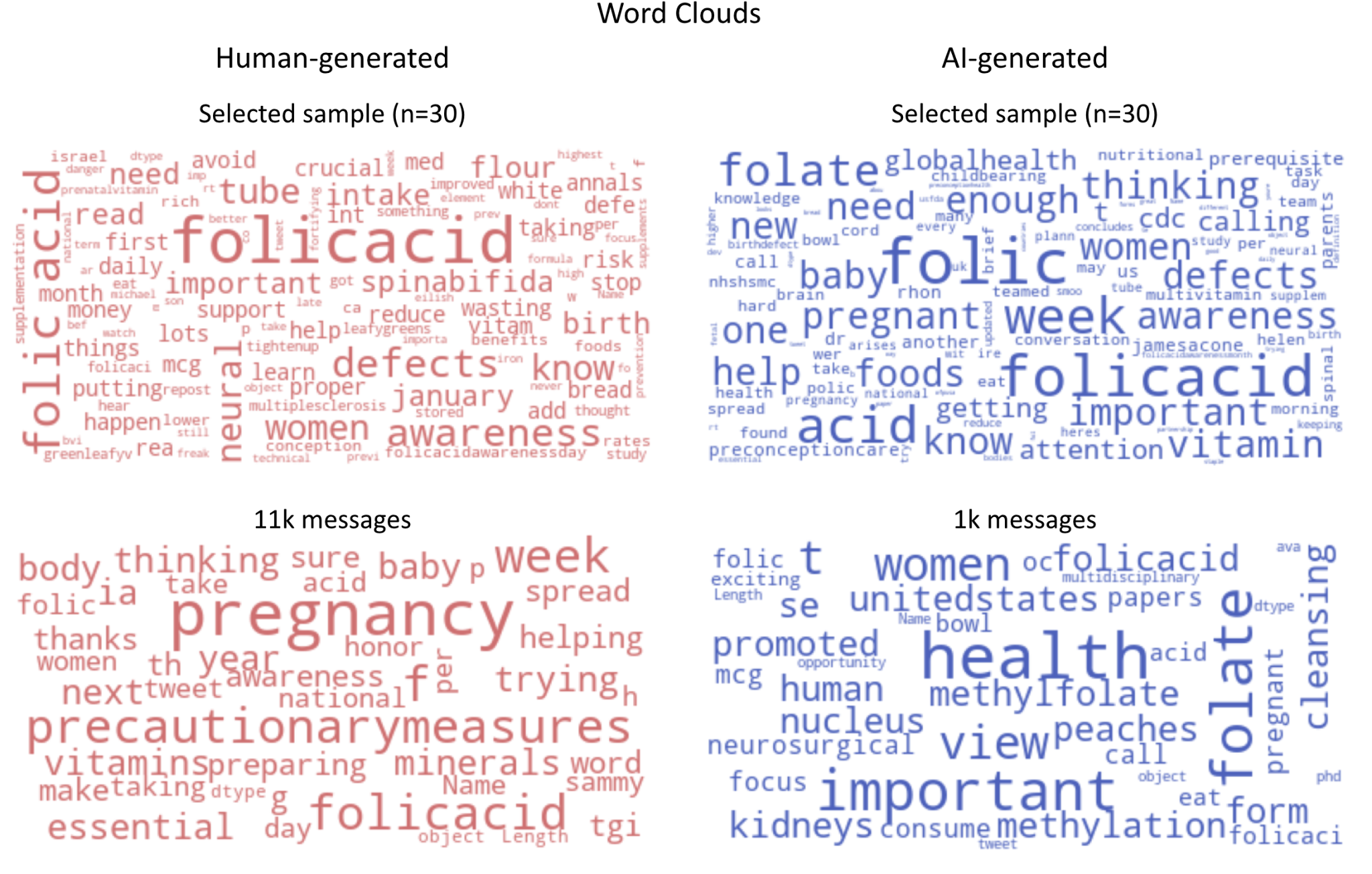


***Supplementary Figure 1:*** *Word clouds for Human- and AI-generated messages. The top row shows word clouds for the selected sample of 30 messages used in the online evaluation, the bottom row illustrates results for the entire sample of 11k human-generated messages and 1k AI-generated messages.*

## **Topic Modeling Results**

Beyond visual inspection and basic frequency analysis, we used topic modeling to explore the semantic structure of the text bodies. In brief, topic modeling treats documents or messages as a mixture of topics, which in turn consist of a mixture of words. Roughly similar to cluster analysis and other unsupervised machine learning techniques, topic modeling identifies latent semantic structure based on co-occurrences of similar words.

Specifically, we used the topicmodels package [[1]](https://paperpile.com/c/L3wDXC/k5NZ) within the R statistical software to compute Latent-Dirichilet-Allocation (LDA) topic models [[2]](https://paperpile.com/c/L3wDXC/mGUC). We used the package ldatuning [[3]](https://paperpile.com/c/L3wDXC/b3b8) to determine a suitable number of topics. By plotting the metrics labelled CaoJuan2009 [[4]](https://paperpile.com/c/L3wDXC/sz25), Arun2010 [[5]](https://paperpile.com/c/L3wDXC/pa5j), and Deveaud2014 [[6]](https://paperpile.com/c/L3wDXC/g6eA) and examining the results, we determined that a 7-topic solution provides a suitable tradeoff between granularity and interpretability. Thus, separate LDA-analysis with k=7 topics were computed for the corpus of Human-generated messages and the AI-generated messages.

An overview of the results is provided in Supplementary Figure 2. As can be seen, the topics are also meaningful in the sense that they are all focusing on issues related to folic acid, such as folic acid & neural tube defects, folic acid & nutrition, folic acid & pregnance, or mandatory fortification of folate-rich food. One noteworthy observation is that for AI-generated messages, topic #3 (green) appears to contain internet-related words. Inspection of this topic suggested that some of the messages within that topic were instances in which the AI-generations appeared to “spill over” reminiscences of the basic GPT2 model, which had been trained on webtext that came from forums, which likely also contained web-related technical terms (e.g. javascript, internet, discussion, etc. cf. [[7]](https://paperpile.com/c/L3wDXC/lr8r).



***Supplementary Figure 2:*** *Topic modeling results. The left part of the figure shows results for Human-generated messages, the right part corresponds to AI-generated messages. The word clouds illustrate top words for each topic based on the beta-value, color-coded for topic. Note that the spatial arrangement of topics is simply circular in order to plot all topic words in one figure; unlike with plots for embeddings, the distance between words does not carry information. The bottom panels illustrate the top words for each topic as bar plots, color-coded in the same schema as the plots on top.*

## **Analysis of semantic similarity**

Lastly, we assessed the semantic similarity of individual messages via the sentence-transformers package [[8]](https://paperpile.com/c/L3wDXC/y2Uy) with the ‘paraphrase-distilroberta-base-v1’ model. To compute the semantic similarity between two sentences, each message is transformed into a sentence embedding, which are then compared via cosine-vector similarity. The approach behind the sentence-transformers package is suitable for this task because it provides fixed-length embeddings that can be compared via e.g. cosine similarity methods, it is computationally highly efficient (requiring only seconds to transform a sentence), and matches the accuracy of state-of-the-art methods.

The resulting similarity matrices between different sentences or messages are illustrated in Supplementary Figure 3. Overall, we find that the messages in the Human- and AI-generated corpora are similar, as can be expected based on the results presented above (e.g. topic models, *N*-Grams).


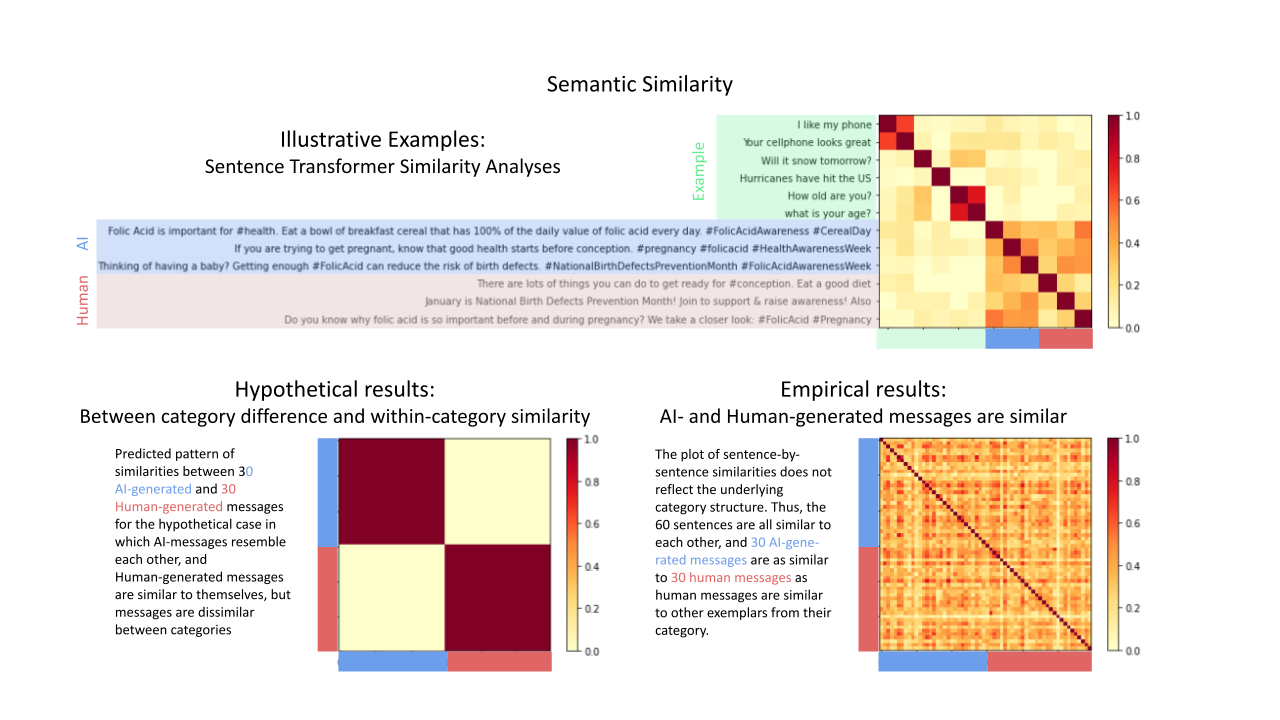


***Supplementary Figure 3:*** *Semantic similarity analysis. Top panel: Six example sentences (green) are presented to illustrate the idea that similar sentence meaning will be reflected in a block-like structure in the similarity matrix plots. Six additional messages (3 AI-generated and 3 Human-generated) are shown to further illustrate the idea that these sentences are quite similar to each other, but different from the example sentences. Bottom left panel: If the AI- and Human-generated messages were only similar to each other, but different across these categories, we would expect a block-like pattern in which 30 AI-messages and 30 Human-generated messages each exhibit high within-category similarity, but low between-category similarity. Bottom right panel: Empirical results of similarity analyses demonstrate that all messages - whether AI- or Human-generated - are quite similar to each other and no immediate between category differences can be seen.*

Specifically, we compared the set of 60 messages (30 AI-generated and 30 human-generated) against each others, and within and across groups. Across all messages, the average similarity was *s* = 0.35. Within the 30 AI-generated messages, the average similarity was *s_AI_* = 0.37 with each other, the average similarity between the 30 human generated messages was *s_Human_* = 0.34. The average similarity between AI-vs.-human messages was *s_AI-vs.-Human_* = 0.35. Testing for differences between these computational indices of semantic similarities revealed no significant differences in any comparison (AI-vs.-Human, within-vs.-across classes; all *p’s* > 0.08).

**References**

1. [Hornik K, Grün B. topicmodels: An R Package for Fitting Topic Models. Journal of Statistical Software. 2011;40: 1–30.](http://paperpile.com/b/L3wDXC/k5NZ)

2. [Blei DM, Ng AY, Jordan MI. Latent dirichlet allocation. the Journal of machine Learning research. 2003;3: 993–1022.](http://paperpile.com/b/L3wDXC/mGUC)

3. [Nikita M. ldatuning: Tuning of the latent dirichlet allocation models parameters. R package version 0 2-0, URL https://CRAN R-project org/package= ldatuning. 2016.](http://paperpile.com/b/L3wDXC/b3b8)

4. [Cao J, Xia T, Li J, Zhang Y, Tang S. A density-based method for adaptive LDA model selection. Neurocomputing. 2009;72: 1775–1781.](http://paperpile.com/b/L3wDXC/sz25)

5. [Arun R, Suresh V, Veni Madhavan CE, Narasimha Murthy MN. On Finding the Natural Number of Topics with Latent Dirichlet Allocation: Some Observations. Advances in Knowledge Discovery and Data Mining. Springer Berlin Heidelberg; 2010. pp. 391–402.](http://paperpile.com/b/L3wDXC/pa5j)

6. [Deveaud R, SanJuan E, Bellot P. Accurate and effective latent concept modeling for ad hoc information retrieval. Document numerique. 2014;17: 61–84.](http://paperpile.com/b/L3wDXC/g6eA)

7. [Carlini N, Tramer F, Wallace E, Jagielski M, Herbert-Voss A, Lee K, et al. Extracting Training Data from Large Language Models. arXiv [cs.CR]. 2020. Available:](http://paperpile.com/b/L3wDXC/lr8r) <http://arxiv.org/abs/2012.07805>

8. [Reimers N, Gurevych I. Sentence-BERT: Sentence Embeddings using Siamese BERT-Networks. arXiv [cs.CL]. 2019. Available:](http://paperpile.com/b/L3wDXC/y2Uy) <http://arxiv.org/abs/1908.10084>
